# Dr. Heywood, on Diseased Uterus

**Published:** 1810-04

**Authors:** W. C. Heywood

**Affiliations:** Blandford


### *To the Editors of the Medical and Physical Journal.*

GENTLEMEN,

MR. WARDROP, in his excellent work on Fungus Hæmatodes, having observed, that the disease so named, "will probably be seldom met with in the ovarium," I am induced to send you for insertion in the Medical Journal, an account of a dissection, which may perhaps be thought a case of that sort. I have added another account of the appearances on dissection of diseased uterus, which, if you think sufficiently interesting, I will beg you to insert also.

Miss W———, æt. 47, had symptoms of diseased uterus for three years.

On opening the abdomen, the diseased mass was found to occupy almost the whole cavity. The liver and stomach were pressed up against the diaphragm, and the former much lessened in bulk; the uterus was considerably enlarged, and on its upper surface, two tumours were seen connected with the peritoneal coat, by a small peduncle; one the size of a large orange flattened, the other of an oblong form and smaller; when cut into, they appeared to consist of a very firm whitish substance. The ovaria were each about the size of a child's head, and converted into an uniform brown pulpy matter, with cells, containing a dark coloured fluid; a few hydatids were on its external surface. The coats of the uterus were much thickened, but not at all in a schirrous state. The cavity was filled with carnous layers and loose pieces of coagulable lymph, in a very putrid state, but no ulceration whatever; the pressure of the mass had produced a contraction of the rectum.

Mrs. B——, æt. 36, the wife of a surgeon in the neighbourhood, after suffering from symptoms of diseased uterus about three years, was seized with excruciating pains over the whole abdomen, which continued till her death, about fourteen hours after this attack.

Dissection.—On opening the abdomen, the peritoneum was found in the highest state of inflammation. The omentum adhered on the left side. In the cavity was found about a pint of serous fluid, and about eight ounces of very fœtid pus. The uterus was enlarged, but not very considerably; and at its fundus, an ulcer about the size of a shilling had opened with the abdomen. The coats of the uterus were much thickened, but there was no scirrosity. The internal surface of the ulcer was about the size of a crown piece; there was no ulceration at the cervix, as described by Dr. Adams, as the usual seat of malignant ulcer of the uterus; of which this was a decided case.

I am, &c.

W. C. HEYWOOD, M. D.

*Blandford, March 7, 1810.*

